# Supplementary figures and images for: Encephalitozoon cuniculi takes advantage of efferocytosis to evade the immune response
Source: PLoS One. 2021 Mar 5;16(3):e0247658. doi: 10.1371/journal.pone.0247658 (PMC7935246; doi:10.1371/journal.pone.0247658)

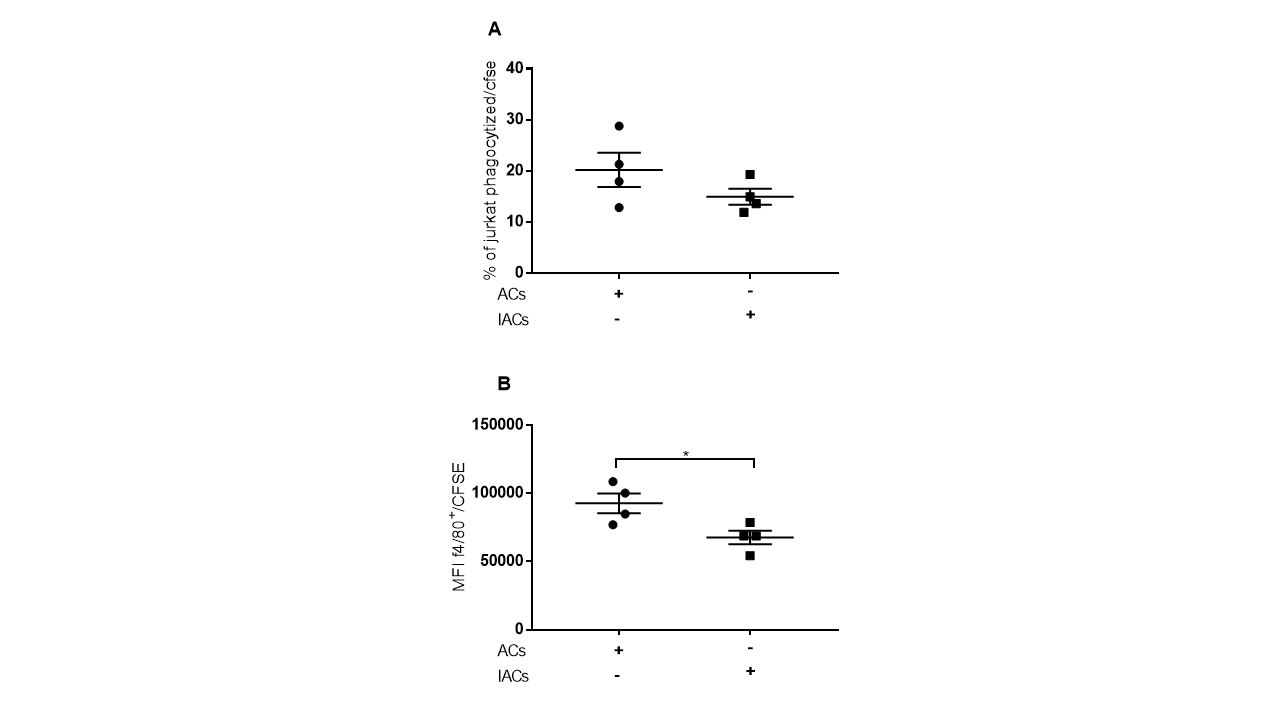

Supplement: S1 Fig — (A) Percentage of phagocytosis of ACs and IACs. (B) Median CFSE fluorescence in macrophages. T-test tested *p < 0.05 with significance between the groups. (TIF) [file pone.0247658.s001.tif]

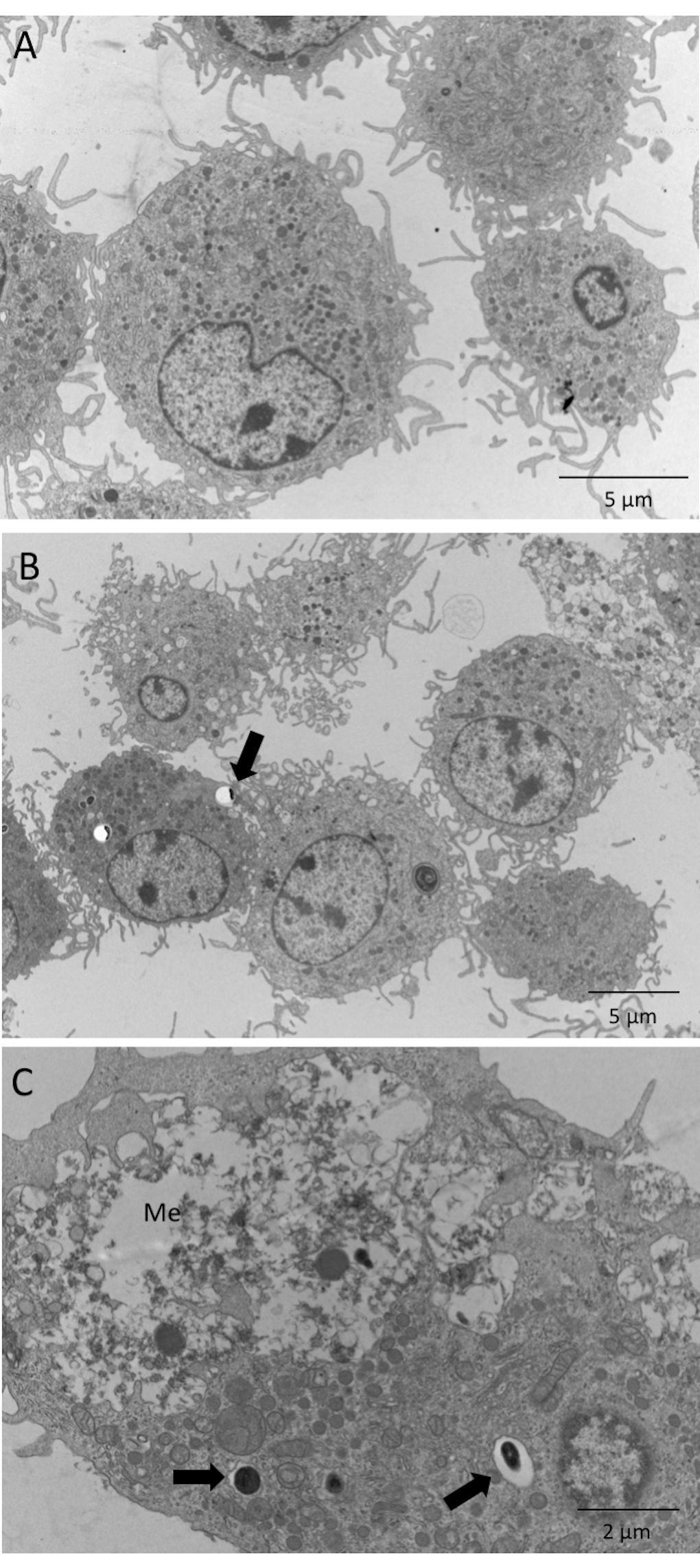

Supplement: S2 Fig — (A) Macrophages controls. (B) Macrophages challenged with E. cuniculi spores show spores internalized in phagosomes in the cytoplasm (arrow). (C) Macrophages with E. cuniculi spores internalized in phagosomes in the cytoplasm (arrow) and with megasomes (Me). (TIF) [file pone.0247658.s002.tif]

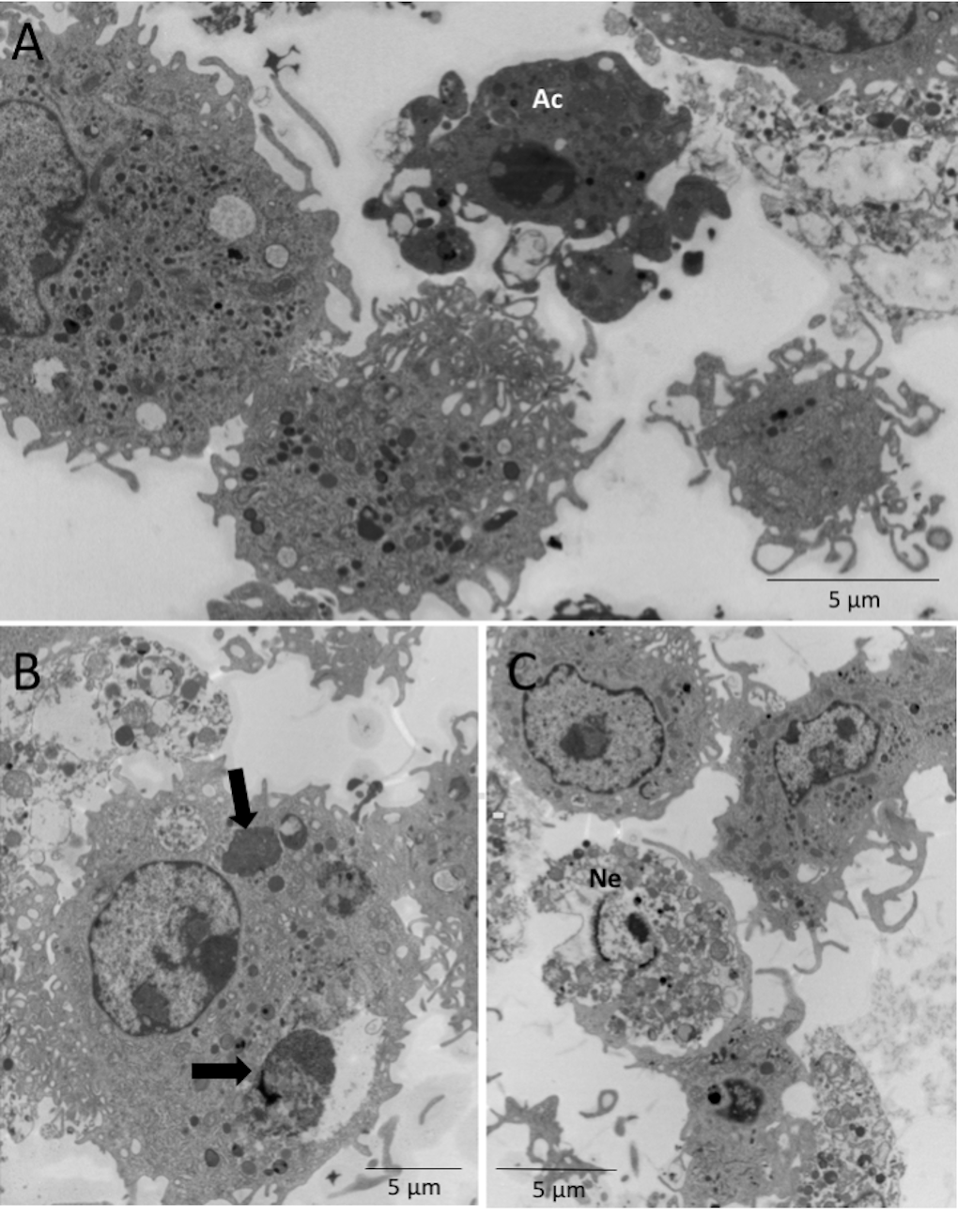

Supplement: S3 Fig — (A) Macrophages around an apoptotic cell (Ab). (B) Apoptotic bodies (arrows) phagocytized by macrophages. (C) A macrophage emitting pseudopods involving necrotic cell content (Ne). (TIF) [file pone.0247658.s003.tif]

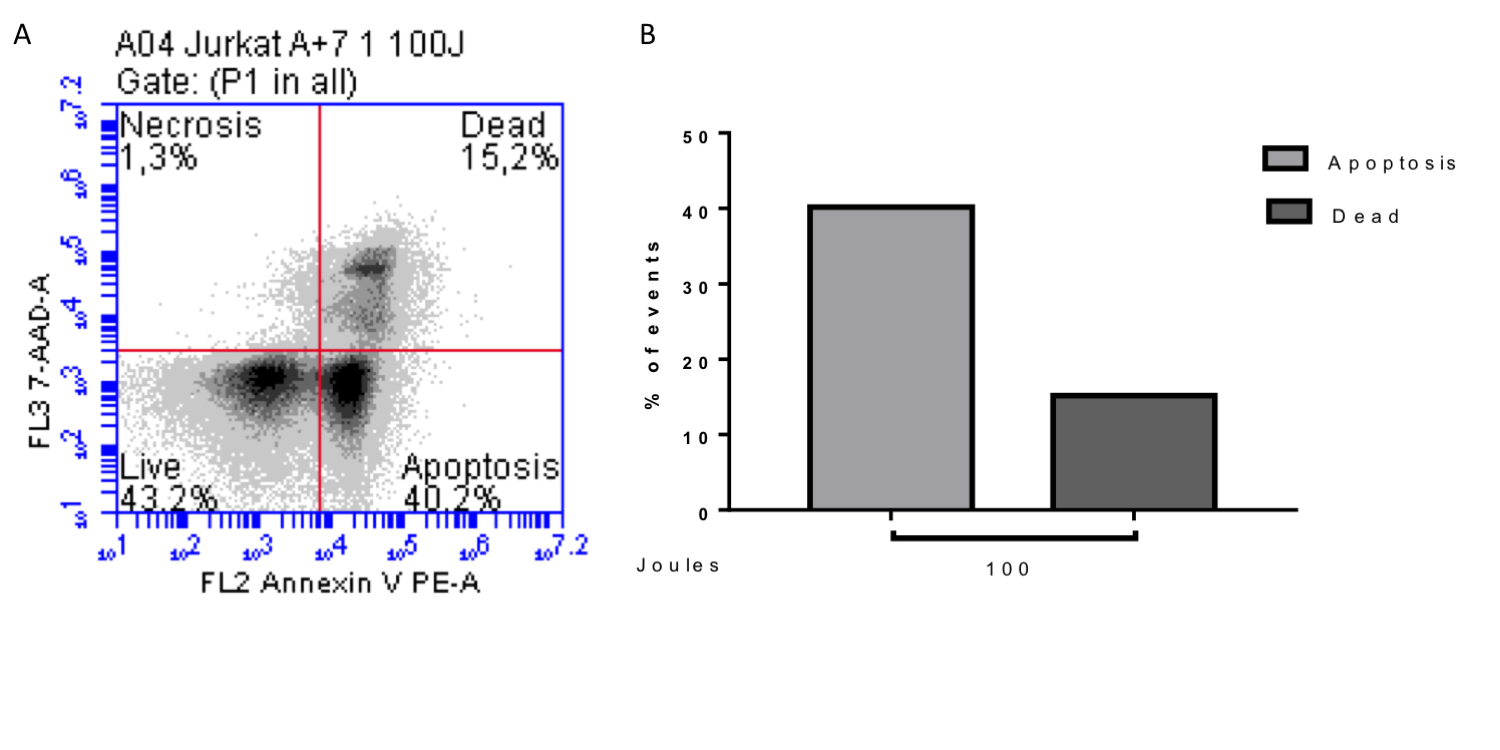

Supplement: S4 Fig — (A) Dot plot showing the percentages obtained from live (7AAD–Annexin–), necrotic (7AAD–Annexin–), apoptotic cells (7AAD–Annexin+), and late apoptosis (7AAD+Annexin–). (B) Percentages obtained from apoptosis (apoptosis) and late apoptosis (death) using 1 pulse with 100 J. (TIF) [file pone.0247658.s004.tif]

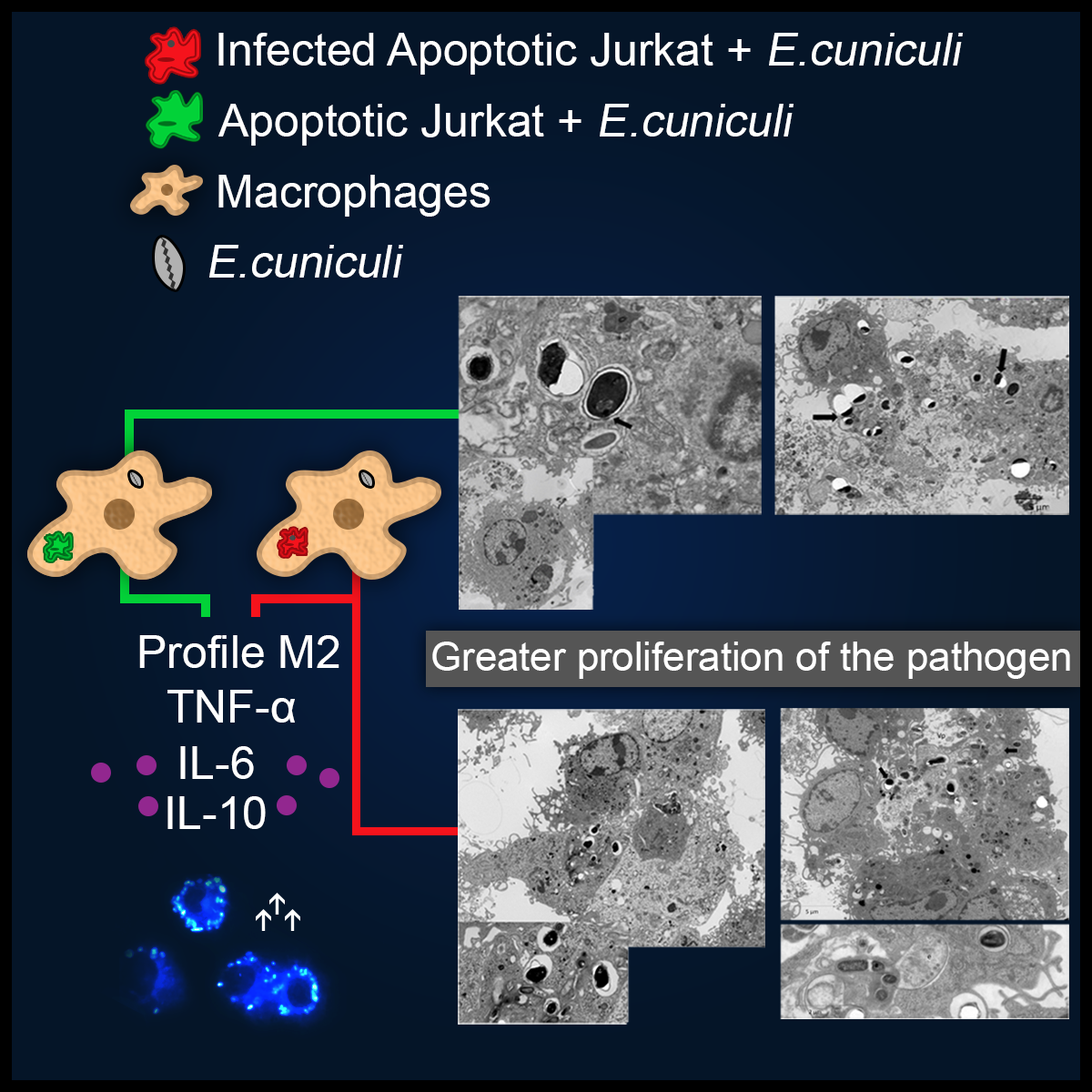

Supplement: S5 Fig — (PNG) [file pone.0247658.s005.png]
